# Correlation between physical characteristics of biopsy specimen and disease of cervical lymph node after contrast-enhanced ultrasound

**DOI:** 10.1186/s12893-022-01671-3

**Published:** 2022-06-11

**Authors:** Wenzhi Zhang, Jie Chu, Gaoyi Yang, Tu Ni

**Affiliations:** Department of Ultrasound, Affiliated Hangzhou Chest Hospital, Zhejiang University School of Medicine (Red Cross Hospital of Hangzhou, Integrated Chinese and Western Medicine Hospital of Zhejiang Province), 310003 Hangzhou, China

**Keywords:** Contrast-enhanced ultrasound, Biopsy, Cervical lymph node, Specimen

## Abstract

**Background:**

To investigate the correlation between physical characteristics and disease of cervical lymph node biopsy specimens after contrast-enhanced ultrasound.

**Methods:**

All patients were biopsied after CEUS, 235 patients were divided into three groups A, B and C according to the physical characteristics of specimens: 92 patients in group A were complete tissue specimens; 113 patients in group B were discontinuous tissue specimens. There were 30 patients in group C, including a small number of tissue and floc, purulent and bloody specimens. Pathological examination, pathogen culture examination and Gene X-Pert MIB examination were completed for all patients in the three groups, and statistical analysis was conducted on the integrity and traits of the specimens.

**Results:**

Group A included 92 intact tissue specimens, 21 with reactive hyperplasia, 17 with lymphoma, 12 with metastatic carcinoma, 13 with lymphadenopathy, 15 with necrotizing lymphadenitis, and rare lymphadenopathy. In group B, 113 patients were treated with intermittent tissue specimens, including infected lymph nodes, lymphoma in 1 case, metastatic carcinoma in 3 cases and sarcoidosis in 1 case. There were 30 patients in group C, including a small amount of tissue and floc, purulent and bloody specimens, all of which were infected lymph nodes. The χ^2^ value of malignant and benign lymph nodes was 42.401, p = 0.000.

**Conclusion:**

The physical characteristics of cervical lymph node biopsy specimens after CEUS are correlated with the disease, which has guiding significance for postoperative specimen selection.

## Introduction

The common causes of cervical lymph node enlargement are cancer, metastasis, infection, reactive hyperplasia, and so on. Ultrasounds guided needle biopsy is widely used in the diagnosis of lymph node diseases [1–5]. Due to the differences in physical properties of specimens, it is often difficult to submit specimens after biopsy, the purpose of this study is to summarize the correlation between the characteristics of specimens after biopsy of enlarged lymph nodes and diseases, so as to provide reference for accurate specimen submission.

## Methods

### Patients

This study was reviewed and approved by the Medical Ethics Committee of Affiliated Hangzhou Chest Hospital of Zhejiang University, and patients gave informed consent. 235 patients with lymph node diseases treated in our hospital from March 2019 to March 2021 were collected and divided into groups A, B and C according to the specimen characteristics after biopsy. Group A were complete tissue specimens. Group B were discontinuous tissue specimens. Group C consisted of a small number of tissue and floccule, purulent and bloody specimens. The pathological specimens of all patients were biopsied by coarse needle, and the pus and blood specimens were extracted by fine needle. All patients in the three groups completed pathological examination, pathogen culture examination and Gene X-Pert MIB examination, and statistical analysis of specimen integrity and traits was conducted.

Inclusion criteria: (1) cervical lymph node enlargement, maximum short diameter of lymph node > 0.5 cm, length to diameter > 1.0 cm; (2) Be above the age of 18; (3) There is no serious cardiopulmonary dysfunction and cannot tolerate needle biopsy; (4) There are visible specimens in the needle groove of puncture biopsy needle: The specimens visible to the naked eye in all the needle groove were group A, Discontinuous specimens or Biopsy needle groove with a sample size greater than 1/3 belonged to group B, The specimens with less than 1/3 of the needle groove or most of them fluid were in group C. Exclusion criteria: (1) In order to reduce allergic reactions and hypersensitivity reactions during the use of SonoVue, patients with a history of allergy to eggs, milk, fish and shrimp were excluded from this study; (2) patients who have taken anticoagulant drugs in the past week; (3) Patients with a history of mental illness who cannot cooperate with complete needle biopsy; (4) Skin damage, skin ulcer and severe psoriasis at the puncture site; (5) Patients whose target lymph nodes are located around blood vessels and cannot be routinely sampled or who have serious bleeding and other complications after sampling.

All participants had given written informed consent and were enrolled in institutional protocols approved by our institutional review board.

### Diagnostic methods

All patients underwent contrast-enhanced ultrasound examination. According to the evaluation of contrast-enhanced ultrasound, the largest lymph node on the affected side or abnormal lymph node with ultrasound was preferentially selected as the target lymph node. Ultrasound-guided puncture biopsy was performed, and all specimens were confirmed by pathology, etiological culture and Gene X-Pert MIB (+),Gene X-PERT MIB is a molecular diagnostic method for tuberculosis in recent years, which can provide diagnostic basis for the diagnosis of lymph node tuberculosis in this study). 94 patients were further confirmed by surgery later.

Contrast-enhanced ultrasonography: (1) The largest lymph node on the affected side or lymph node with abnormal ultrasound appearance were selected as the target lymph node. (2) The L9-3 broadband linear array probe with 3.0–9.0 MHz frequency was used in the ultrasound angiography; pulse-inversion harmonic imaging with a low mechanical index (MI) of 0.06 was used in the imaging. The contrast agent was obtained from SonoVue (Bracco), diluted with 5 ml of physiological saline before use and shaken well, then 2.4 ml of the contrast agent was injected into the uperficial elbow vein via bolus injection, followed by 5 ml of physiological saline. (3) Perfusion enhancement of the whole lymph node was observed for 2 min in real time, then the image of the entire imaging process was stored on the instrument hard disk. The contrast-enhanced ultrasound results were categorized into three types of enhancement: homogeneous, heterogeneous and none. Partial enhanced lymph nodes and uniformly enhanced lymph nodes were selected for ultrasound-guided puncture biopsy, with the enhanced biopsy as the main sampling target area.

### Statistical analysis

Data analysis was performed using SPSS 23.0 statistical software. The measurement data are expressed as the mean ± standard deviation (x ± s) and were compared via t-tests. Countable data were expressed as the rate (%), and the two groups were compared using a χ^2^ test. Diagnostic tests were performed using sensitivity, specificity, positive, and negative predictive values, accuracy, and area under the receiver operating characteristic (ROC) curve. Differences were considered statistically significant at p < 0.05.

## Results

The sex, age and lymph node CEUS mode of patients are shown in Table 1. These patients had a history of malignant tumor in 21 cases, a history of tuberculosis in 80 cases, combined with other system tuberculosis in 27 cases.

**Table 1:** Basic information of patients and CEUS

Group	A (92)	B (113)	C (30)
Sex			
Male	53	37	12
Female	39	76	18
Age range	18–68	19–71	18–59
Average age	46 ± 7.2	44 ± 5.3	37 ± 5.7
Enhanced mode			
Homogeneous enhancement	78	0	0
Heterogeneous enhancement	14	113	30

The comparison of puncture specimen status and disease correlation among patients in the three groups is shown in Table 2.

**Table 2:** The state of puncture specimen is correlated with disease

Group	A	B	C
Number of cases	92	113	30
Lymphoma	17	1	0
Metastatic carcinoma	12	3	0
Reactive hyperplastic	21	0	0
Tuberculous lymphadenitis	13	96	21
Non-tuberculous mycobacteria	0	3	2
Fungal lymphadenitis	0	2	1
Common bacterial lymphadenitis	0	1	6
necrotic lymphnoditis	15	5	0
Castleman	5	0	0
Kimura’s disease	2	0	0
PAWS disease	2	1	0
sarcoidosis	3	1	0
Langerhans cell hyperplasia	1	0	0
plasmacytoid blastoid dendritic cell tumor	1	0	0
Percentage (%)	39.1	48.1	12.8

Comparison of physical characteristics of malignant and benign lymph node specimens is shown in Table 3.

**Table 3:** Correlation of lymph node specimens between malignant and benign groups

Group	Number of cases	Malignant lymph node	Benign lymph node
A	92	31	61
B	113	4	109
C	30	0	30
Χ^2^	42.401		
P	0.000		

## Discussion

**Fig. 1:**
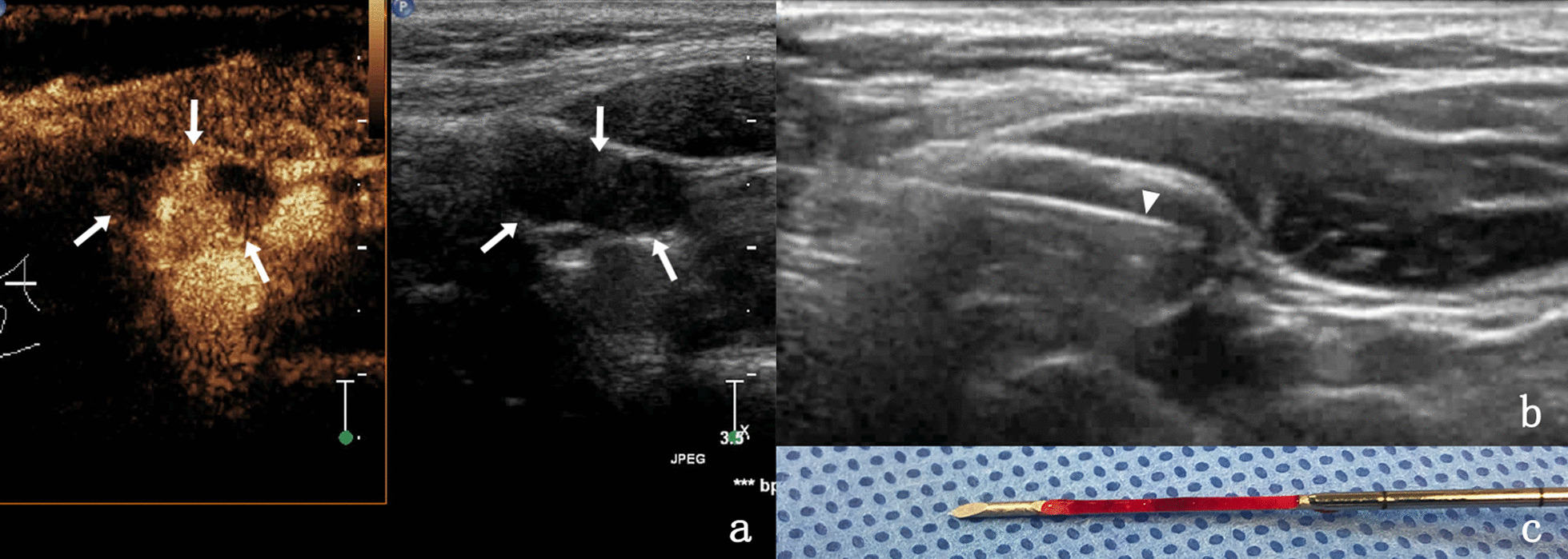
**a** Lymph node enlargement in ipsilateral neck region II was found in a 31-year-old female one year after surgery for thyroid cancer, which was not clearly diagnosed by two biopsy in other hospitals. After CEUS evaluation, heterogeneous enhancement of lymph nodes was found, with enhancement area in the middle (indicated by the arrows). **b** For the same patient, needle biopsy was performed on the enhanced area under the guidance of CEUS, and the triangle arrow is needle biopsy. **c** Although the specimen was broken tissue and bloody fluid, the pathological examination showed papillary thyroid carcinoma metastasis, so it was a valid specimen

The causes of superficial lymphadenopathy are various, Table 1 only lists the pathological types of lymph node diseases in our hospital from March 2019 to March 2021. Clear the causes of enlarged lymph nodes can not only guide the clinical treatment, also is very important to the prognosis of disease, at present the gold standard for lymph node disease diagnosis lymph nodes after surgical biopsy pathology diagnosis [3, 6–8], Surgical biopsy is very damaging to the patient, plus some lymph nodes around the complex organization structure, position, deep or factors such as small volume, Results in difficult surgical biopsy and high surgical risk. Compared with surgical biopsy, ultrasound-guided puncture biopsy has less trauma, faster recovery, convenient operation and can significantly reduce the pain of patients. Currently, ultrasound-guided puncture biopsy after CEUS is considered as a scientific method to quickly obtain effective pathological tissues under non-surgical conditions (Fig. 1).

In order to improve the integrity of biopsy specimens, this study used CEUS combined with puncture biopsy. CEUS can accurately display the active components and necrotic areas in lymph nodes. The integrity specimen rate was about 39.1% (group A), and the rate of discontinuous specimen was about 48.1% (group B). The specimen rate of small amount of tissue and floccule, purulent and bloody was 12.8% (group C), and the effective sample rate of selected pathological examination was 87.2% (group A and B), which was consistent with the report [9]. The proportion of malignant lymph nodes in cases with complete specimens was high, while the proportion of benign disease was higher in intermittent specimens and fluid specimens, indicating that specimen integrity and physical properties after puncture biopsy were correlated with diseases.

There was a certain correlation between the proportion of enhanced area in lymph nodes and the disease. The integrity of cervical lymph node biopsy specimens was directly related to the proportion of enhanced area in lymph nodes, and homogeneous enhanced lymph nodes had higher specimen integrity. In this study, the specimens integrity of malignant lymph nodes after CEUS combined with puncture biopsy was higher than that of benign lymph nodes, The reason is that tumor cells and lymphoma cells in metastatic lymph nodes are hyperplasia and active, leading to high tension and hard texture in the lymph nodes. After biopsy, the specimens are usually complete strips. When there is necrosis in the lymph nodes, it is usually coagulated necrosis or blood vessel rupture and hemorrhage to form hematoma [10], showing no enhancement, which may affect specimen integrity. For patients with metastatic lymph nodes of nasopharyngeal carcinoma and thyroid carcinoma, preoperative CEUS usually shows most of the non-enhanced areas in lymph nodes [11, 12], which cannot be sampled by histological biopsy, and fine needle aspiration is full blood fluid. Therefore, lymph nodes with high enhancement ratio beside it should be selected for sampling, which can meet the requirements of pathological examination with higher specimen integrity [10]. In this study, 3 mainly cystic metastatic lymph nodes were 2 metastatic lymph nodes of nasopharyngeal carcinoma and 1 metastatic lymph node of thyroid carcinoma. The cystic part was old bloody fluid under ultrasound-guided aspiration. Benign lymph node specimens integrity in group B, but lymph node reactive hyperplasia for benign lesions, after biopsy are complete specimen, mainly because of its pathology can be characterized by enlarged lymph node follicle and increase in the number, also can show significant enlargement of the paracortical or interfollicular areas, hyperplasia of cells diversification, postcapillary venules with high endothelial often proliferate [13], Vascular embolism and liquefaction necrosis were reported in rare reactive hyperplasia of lymph nodes.

Some studies have confirmed that lymphoma rarely occurs necrosis, usually caused by trophoblast artery embolization resulting in coagulative necrosis. CEUS can intuitively show the necrotic area in lymphoma [10, 14–17], coagulative necrosis was described in the pathologic findings of lymphoma cases with intermittent specimens in this study.

**Fig. 2:**
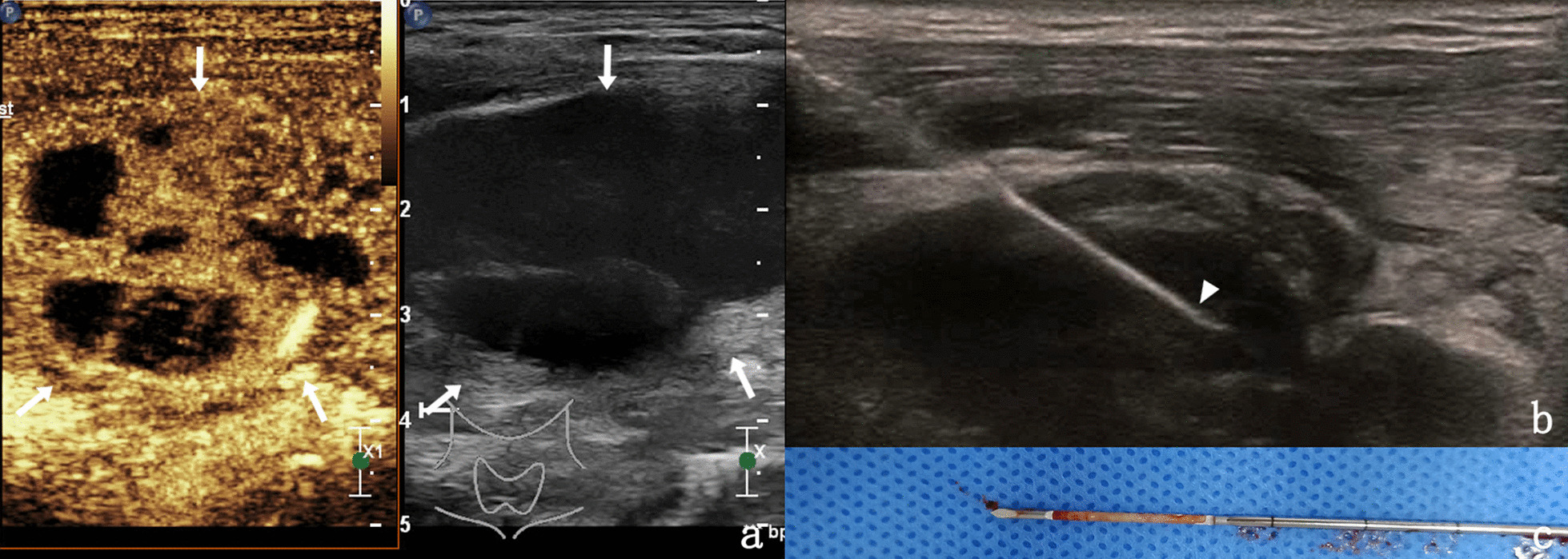
**a** A 25-year-old male with cervical lymph node tuberculosis was evaluated by CEUS (indicated by the arrows), which revealed heterogeneous and septal enhancement of lymph nodes. **b** Needle biopsy was performed on the enhanced area under CEUS guidance, with biopsy needle at triangle arrow. **c** After biopsy, the specimen was intact in strips

**Fig. 3:**
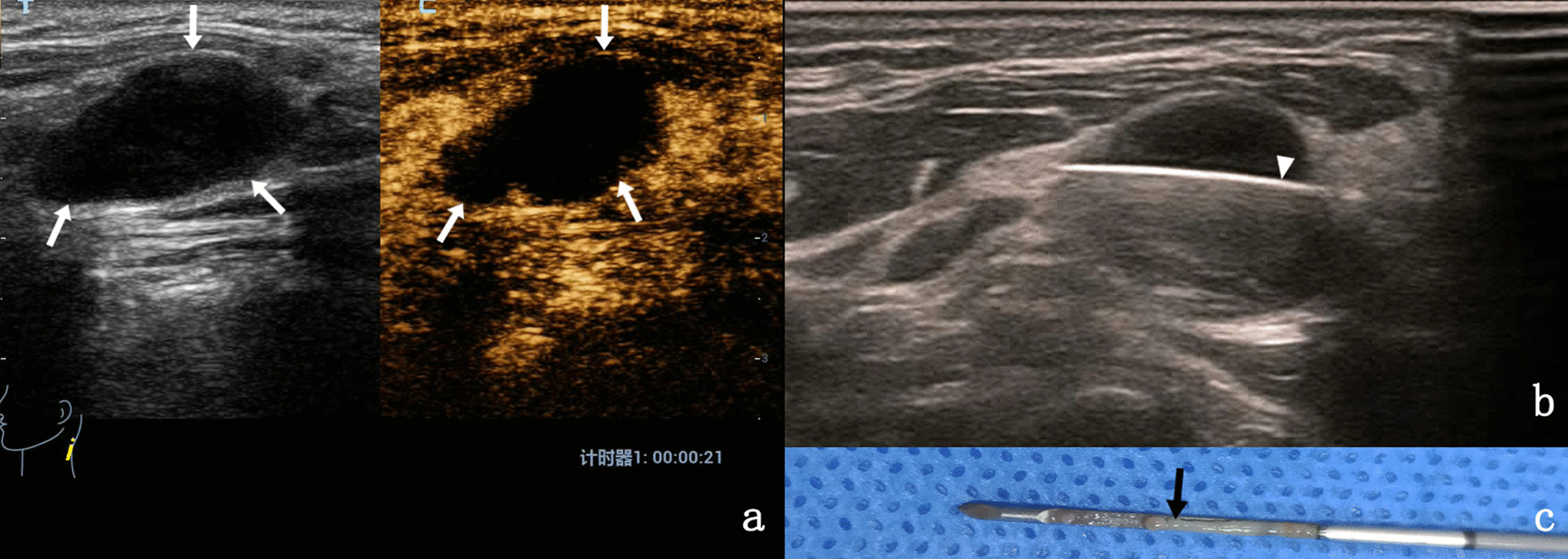
**a** A 45-year-old woman with cervical lymph node tuberculosis was evaluated by CEUS, which revealed heterogeneous and annular enhancement of lymph nodes (indicated by the arrows). **b** Under the guidance of CEUS, the puncture Angle was adjusted to perform the puncture biopsy, and the biopsy needle is indicated by the triangle arrow. **c** The specimen is broken tissue with necrotic material (arrow)

There is a great difference in specimen integrity in lymph node infectious diseases (Figs. 2, 3), which may be related to the virulence of pathogenic microorganisms, and its CEUS manifestations are mainly heterogeneous enhancement and annular enhancement [18, 19]. The infectious diseases in this study were mainly lymph node tuberculosis, which may be related to the fact that our hospital is a special tuberculosis hospital, or it may be related to the fact that it is a chronic process, which is often ignored by patients and significantly enlarged at the later stage, while common bacterial infection lymph node inflammation is obvious, early treatment and early use of effective antibiotics。

In this study, necrotizing lymphadenitis, sarcoidosis and other benign lymph node specimens accounted for about 75%, and most cases were located in group A. The integrity of specimens was related to the scope of necrosis. The number of sarcoidosis cases in this study was small, and the integrity of specimens needs to be further studied.

For rare lymph node diseases, such as Castleman, Langerhans cell hyperplasia and plasmacytoid blastoid dendritic cell tumor, the puncture specimens in this study are all complete specimens, which are located in group A. Castleman is A hyperplastic disease, and its high integrity is related to its pathological state. The latter two are neoplastic. High integrity may be related to this. Kimura disease is a rich vascular endothelial cell hyperplasia, and a large lymphocyte, eosinophil infiltration of inflammatory lesions [19], and lymph node involvement of lymph node structure, with the majority of lymphoid follicles, there is a lot of eosinophilic cells between follicular, increase blood vessels is a proliferative disease [20], in this study, 2 cases were complete specimen Its high integrity may be related to the above pathological characteristics, but overall the number of cases of the above diseases is not enough, which needs to be further confirmed by increasing the sample size.

In conclusion, the integrity of specimens after CEUS combined with biopsy is correlated with diseases to a certain extent. Specimens with high integrity have a high proportion of malignant lymph nodes, which may also be benign diseases such as reactive hyperplasia. CEUS shows homogeneous enhancement. Intermittent and liquid specimens are more likely to be benign lymph nodes. For example, in infectious diseases, specimen submission needs to be diversified, and most of its CEUS manifestations are heterogeneous enhancement and annular enhancement.

## Data Availability

All data generated or analysed during this study are included in this published article.
